# Supplementary material for: MetaRibo-Seq measures translation in microbiomes
Source: Nat Commun. 2020 Jun 29;11:3268. doi: 10.1038/s41467-020-17081-z (PMC7324362; doi:10.1038/s41467-020-17081-z)
Supplement: Supplementary file 10 — Supplementary Data 7 [file 41467_2020_17081_MOESM10_ESM.zip › File2/Confidence_High_Taxonomy/132754_out.krona.html]

Javascript must be enabled to view this page.

members
magnitude
magnitudeUnassigned
count
unassigned
taxon
rank

132754\_out

19

19
superkingdom
2

976
phylum
19

class
200643
19

19
171549
order

19
171550
family

239759
genus
19

19

SRS011405\_contig\_number\_18302SRS015960\_contig\_number\_4441SRS016095\_contig\_number\_12890SRS016335\_contig\_number\_48103SRS020328\_contig\_number\_39136SRS024009\_contig\_number\_contig-100\_886.86509SRS045826\_contig\_number\_11753SRS046502\_contig\_number\_10245SRS046717\_contig\_number\_6081SRS054905\_contig\_number\_24887SRS056259\_contig\_number\_27560SRS075773\_contig\_number\_contig-100\_1742.240461SRS077194\_contig\_number\_12231SRS1055083\_contig\_number\_contig-100\_7036.7037SRS142890\_contig\_number\_41685SRS143466\_contig\_number\_5898SRS143523\_contig\_number\_17550SRS143876\_contig\_number\_contig-100\_688.232836SRS148424\_contig\_number\_3325
214856
species
